# Supplementary material for: Thermodynamic-driven polychromatic quantum dot patterning for light-emitting diodes beyond eye-limiting resolution
Source: Nat Commun. 2020 Jun 16;11:3040. doi: 10.1038/s41467-020-16865-7 (PMC7297963; doi:10.1038/s41467-020-16865-7)
Supplement: Supplementary file 1 — Supplementary Information [file 41467_2020_16865_MOESM1_ESM.pdf]

Supplementary Information

Thermodynamic-driven polychromatic quantum dot patterning for light-emitting diodes beyond eye-limiting resolution

Tae Won Nam,[†] Moohyun Kim,[†] Yanming Wang,[‡] Geon Yeong Kim,[†] Wonseok Choi,[†] Hunhee Lim,[†] Kyeong Min Song,[†] Min-Jae Choi,[†] Duk Young Jeon,[†] Jeffrey C. Grossman, [‡] and Yeon Sik Jung*,[†]

[†]Department of Materials and Science and Engineering, Korea Advanced Institute of Science and Technology, 291 Daehak-ro, Yuseong-gu, Daejeon 305-701, Republic of Korea

[‡] Department of Materials Science and Engineering, Massachusetts Institute of Technology, Cambridge, Massachusetts 02139, United States

Contact information

Corresponding Author

*,[†]E-mail ysjung@kaist.ac.kr (Y.S.J.)

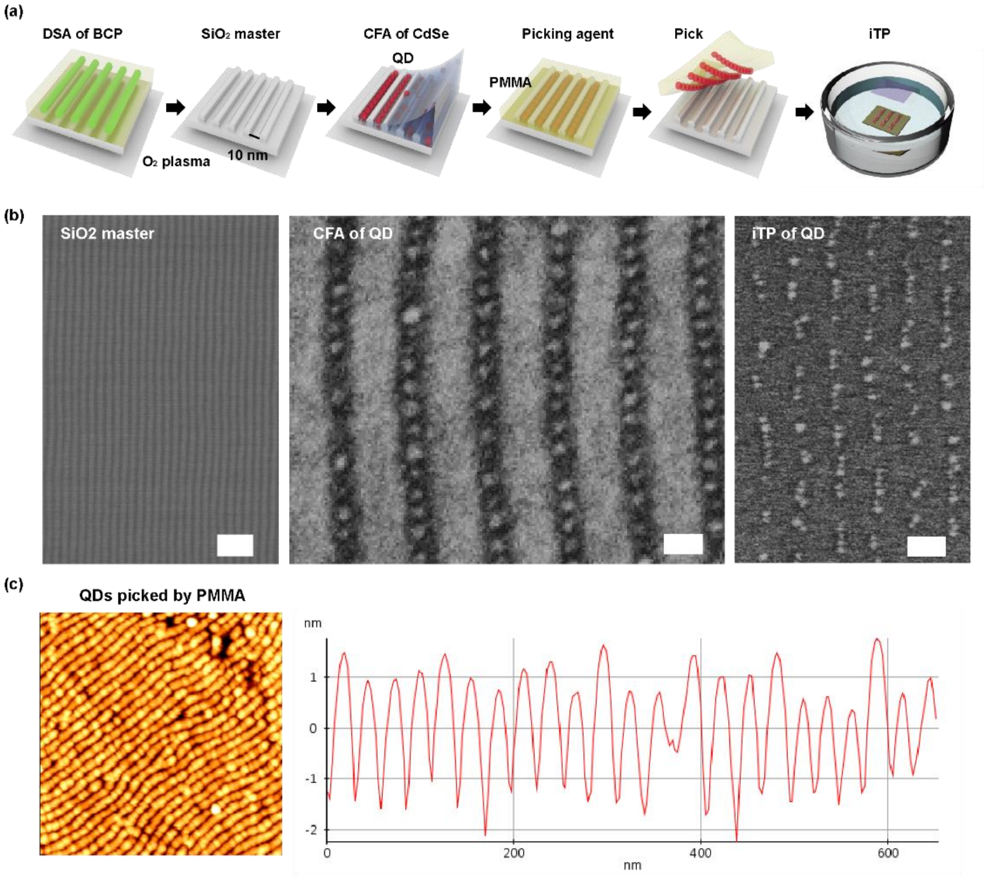

Supplementary Figure 1 | Single-QD-width array self-assembly and iTP. (a) Schematic of self-assembly and iTP process of single-QD resolution array. A hard master template is prepared by PS-*b*-PDMS BCP self-assembly. **(b)** SEM images showing overall single-QD-width array fabrication. Sub-20 nm, BCP-based hard master template (left). Self-assembled QDs in the master template (middle). Single-QD array on target Si substrate using iTP (right). **(c)** AFM image of picked QDs using PMMA transfer medium (left) and corresponding height profile (right).

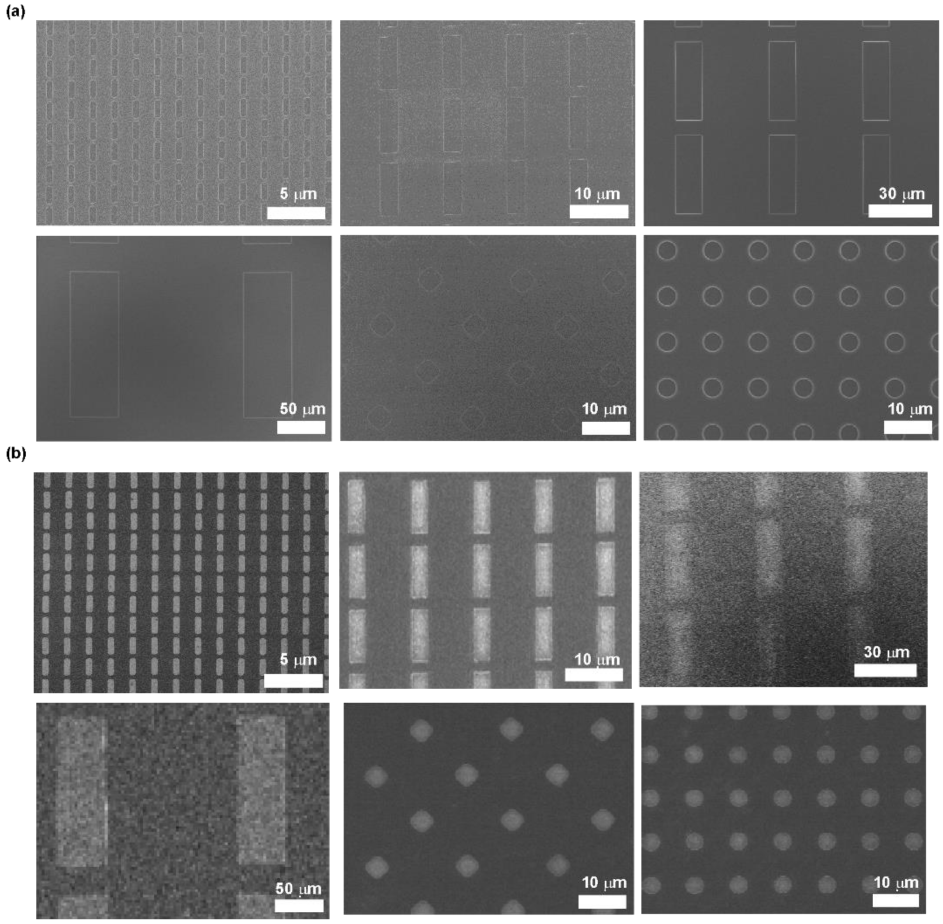

Supplementary Figure 2 | Application-specific template design and iTP of QD arrays. (a) SEM images showing Si master template fabricated by KrF photolithography process and subsequent plasma etching. The resolution of the templates range from 500 nm to 50 μm. Diamond and circular trenches are prepared for hexagonal pentile QD arrays. **(b)** SEM images of iTP QD arrays corresponding to the master templates in (a).

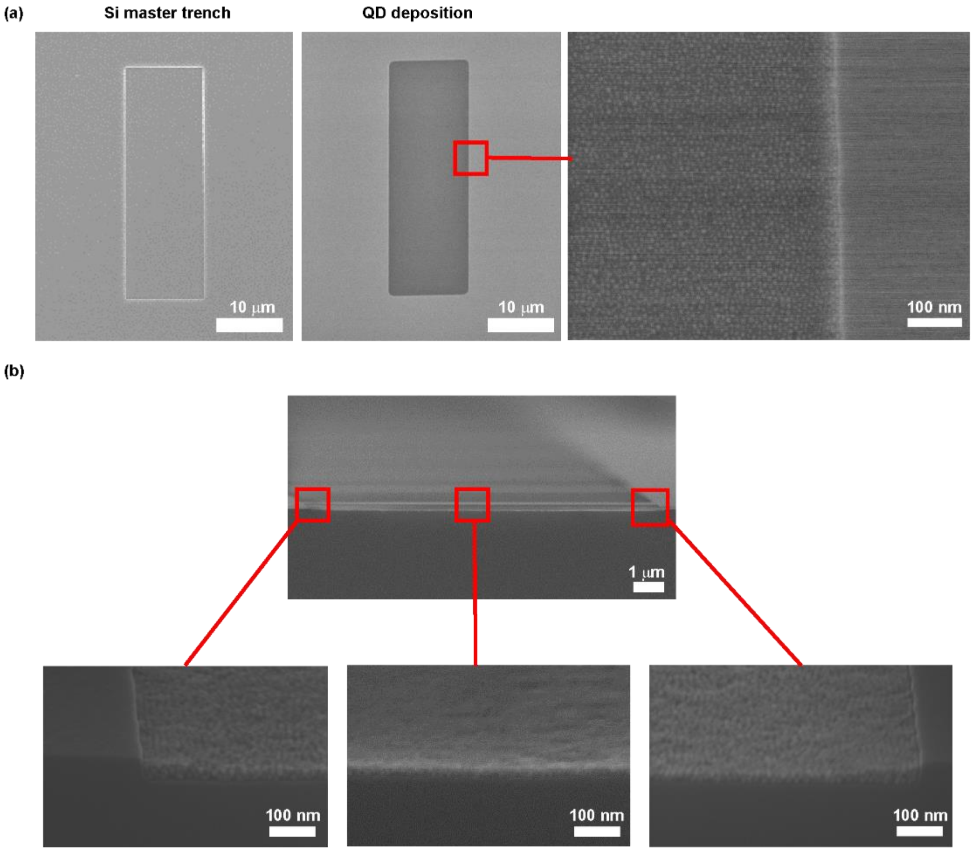

Supplementary Figure 3 | Thickness uniformity of QD patterns (a) Top-down SEM images of an empty Si master trench (left) and QD patterns assembled in the trench (middle and right). **(b)** Cross-section SEM images of QD-deposited trench (top). Higher-magnification SEM images for each edge (bottom left and right), and centre (middle).

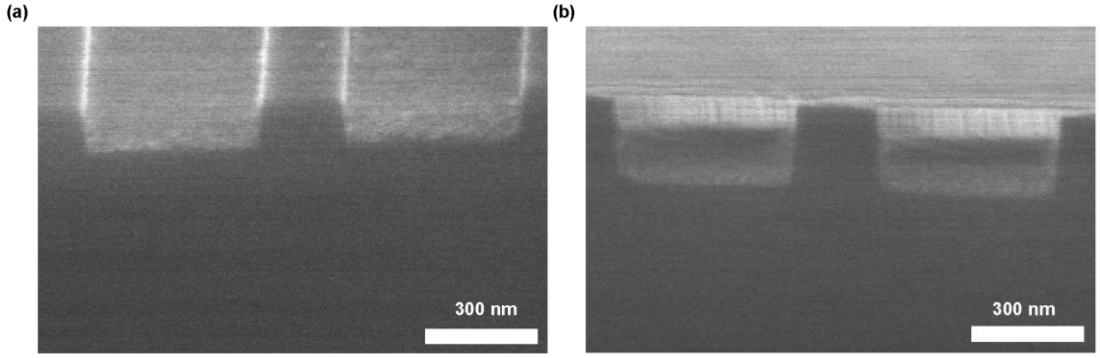

Supplementary Figure 4 | Control of assembled QD film thickness (a) Tilted and (**b**) cross-section SEM images of 150 nm thick patterned QD films using 250 nm depth trench template.

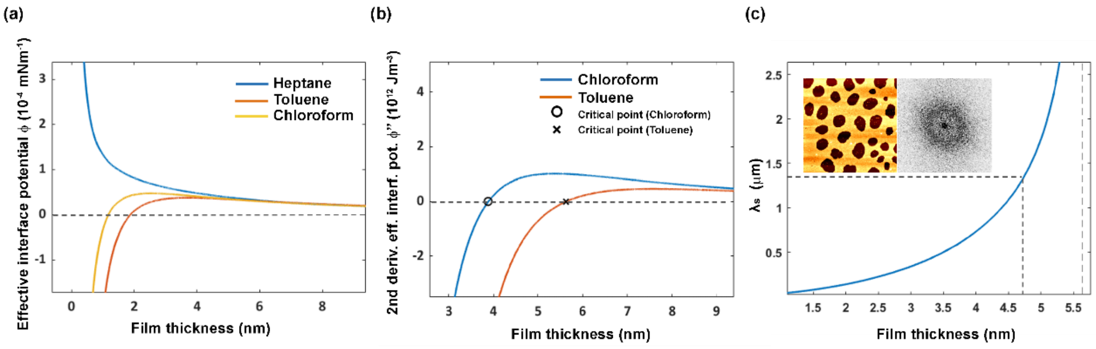

Supplementary Figure 5 | Solvent stability analysis via effective interface potential calculation. (a) The calculated effective interface potential of heptane, toluene, and chloroform liquid film in Si/SiO_x/PDMS/solvent/air regime is plotted as a function of the solvent film thickness. **(b)** The second derivatives of effective interface potential of chloroform and toluene are plotted as a function of the solvent film thickness. The critical points marked as O and X indicate the critical film thickness, h_{crit}, under which spinodal dewetting occurs. **(c)** The calculated characteristic wavelength λ_s of toluene is plotted as a function of the solvent film thickness. AFM image (left) and corresponding FFT image (right) of dry QD film spun-cast under binary solvent composition regime are shown in the inset.

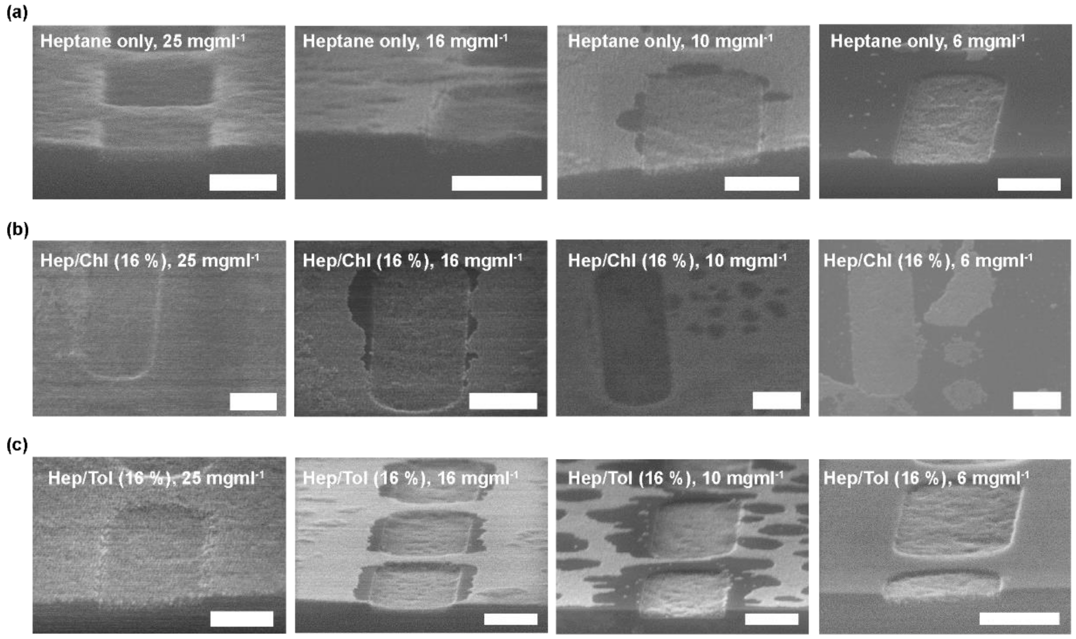

Supplementary Figure 6 | QD concentration and solvent composition dependent QD film morphology. SEM images showing QD film morphologies of **(a)** heptane only, **(b)** heptane/chloroform (16% chloroform), and **(c)** heptane/toluene (16% toluene) solution at different QD concentrations. The scale bars denote 300 nm. The scale bars denote 300 nm.

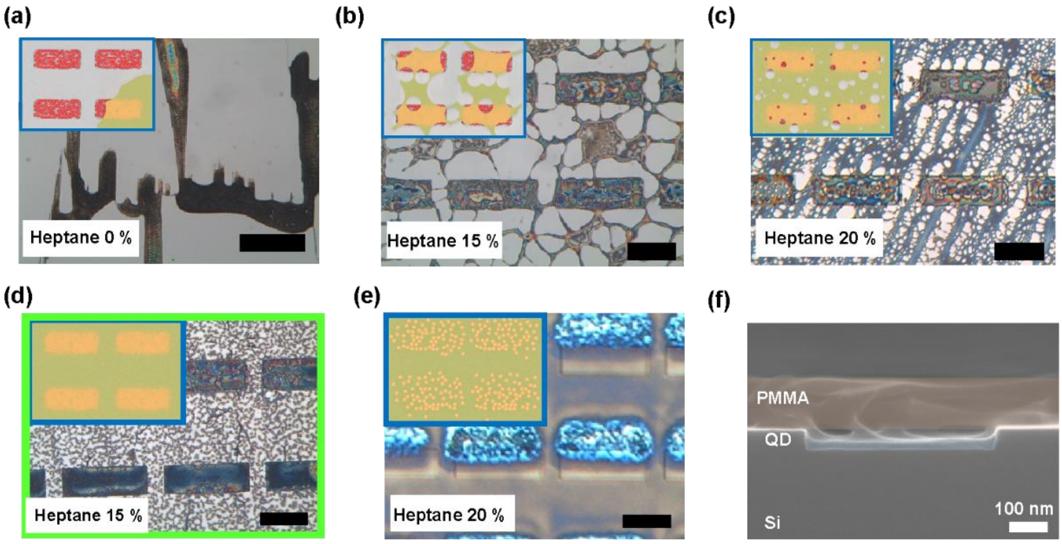

Supplementary Figure 7 | Solvent composition-dependent wetting behavior of PMMA transfer-media. (a-e) Optical images of spun-cast PMMA film on patterned QD substrate at different heptane composition are shown. Schematics of each images are shown in respective insets. Scale bar of (a) denotes 100 μm. Scale bars of (b-e) denote 5 μm. **(f)** Cross-section SEM image of PMMA transfer layer spun-cast on the QD-patterned Si. The patterned QD film is shadowed in blue and the deposited PMMA layer in red.

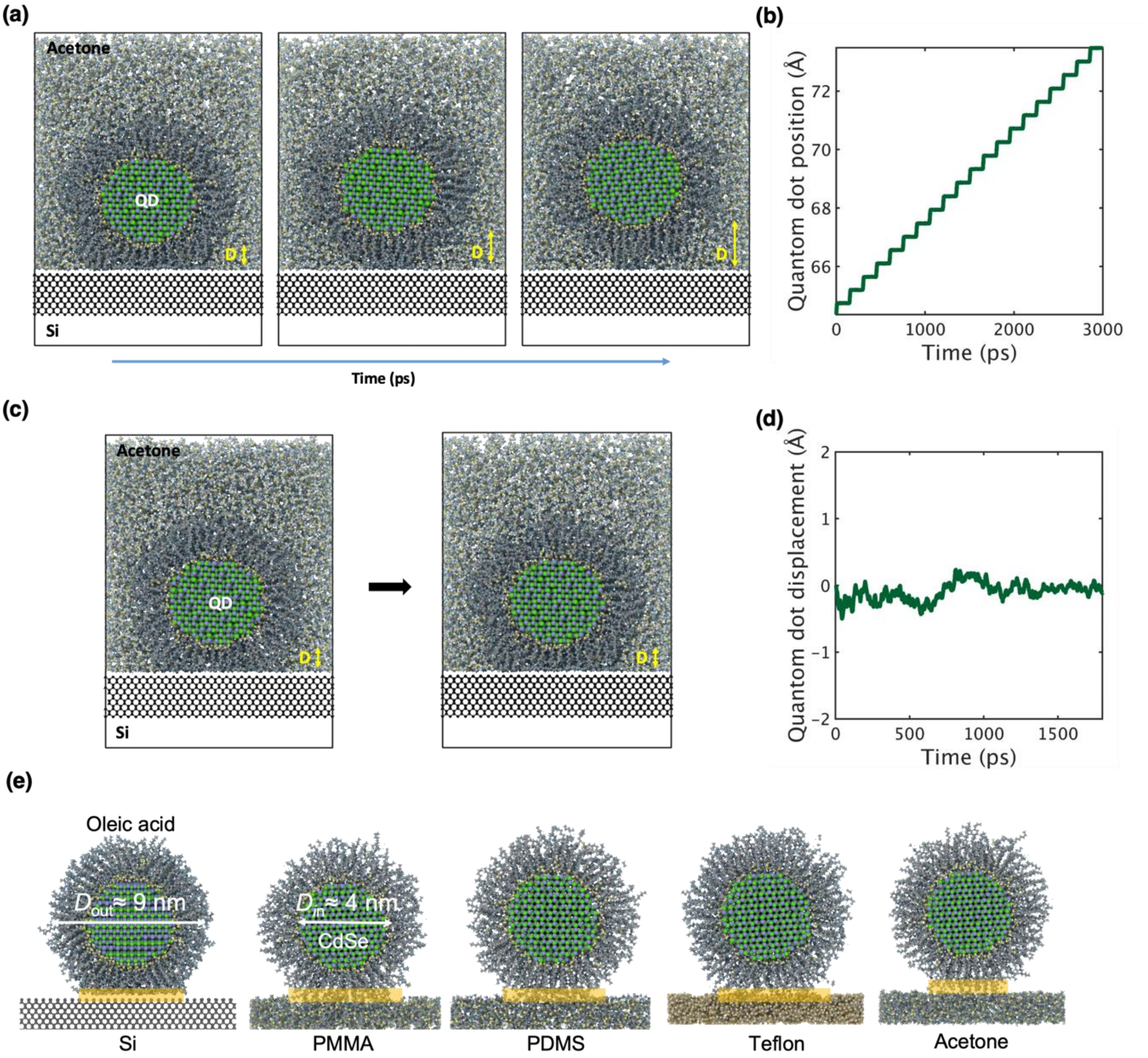

	QD-Si	QD-PMMA	QD-PDMS	QD-Teflon	QD-Acetone
Averaged QD-substrate interaction energy (Kcal/mole)	176.17	163.99	108.17	102.41	53.44
Standard deviation (Kcal/mole)	4.67	7.68	6.93	6.07	3.65
Contact area (nm^2)	25.97	24.17	19.28	19.35	17.64
Averaged energy per area (mJ/m^2)	47.07	47.08	38.93	36.72	21.02

Supplementary Figure 8 | MD simulation results supporting the iTP mechanism. (a) MD simulation results for the change in vertical displacement D, of a QD from a Si surface. The factitious change in the QD position as a function of time is plotted in **(b)**, and the excess energy necessary to lift the QD to each position is plotted as shown in Fig. 4(c). **(c)** MD simulation result to support the stability of a QD on a Si surface surrounded by acetone molecules. The change in the displacement of the QD as a function of time is plotted in **(d)**. **(e)** MD simulation results to calculate the hypothetical interaction energy between each QD-media interface. The extracted interaction energy values are summarized in the table (bottom). The quantity of averaged interaction energy per area was used to plot Fig. 4(g).

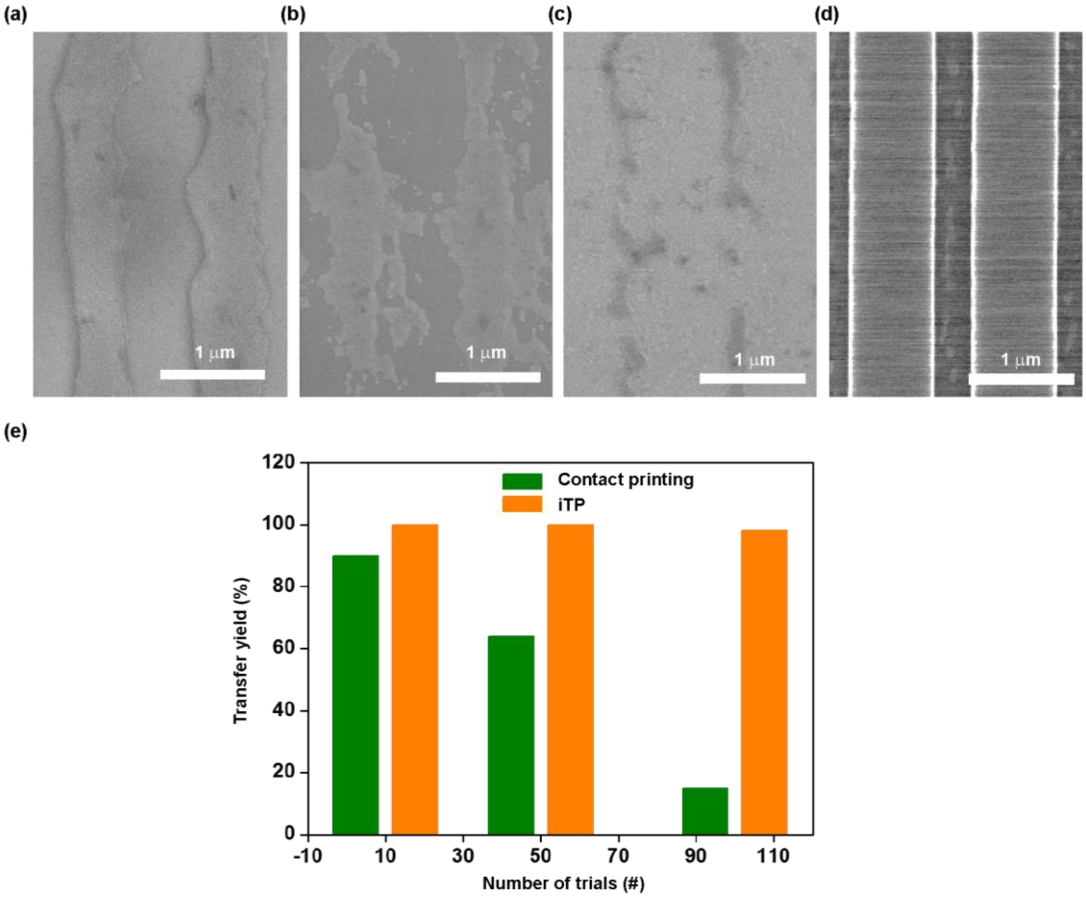

Supplementary Figure 9 | Contact-printed QD pattern vs iTP QD pattern. (a-c) SEM images of printed QD line pattern after 10 (a), 50 (b), and 100 (c) repeated printing cycles using PDMS-elastomer-based contact-printing. **(d)** SEM image of printed QD line pattern after 100 repeated printing cycles using iTP. **(e)** Transfer yield of QD patterns using structured stamping and iTP after repeated printing cycle.

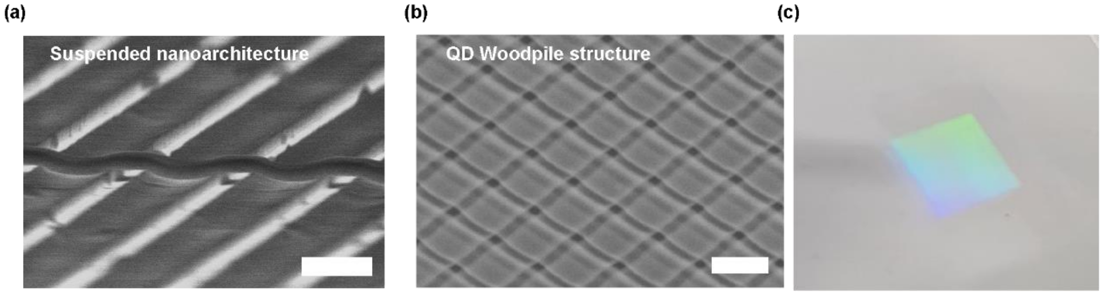

Supplementary Figure 10 | Fabrication of 3D QD woodpile structure using suspended architecture. (a) Tilted-SEM image of 3D QD suspended architecture fabricated using iTP. **(b)** SEM image of 3D QD woodpile structure fabricated using multiple stack printing of iTP. The scale bars denote 1 μm.

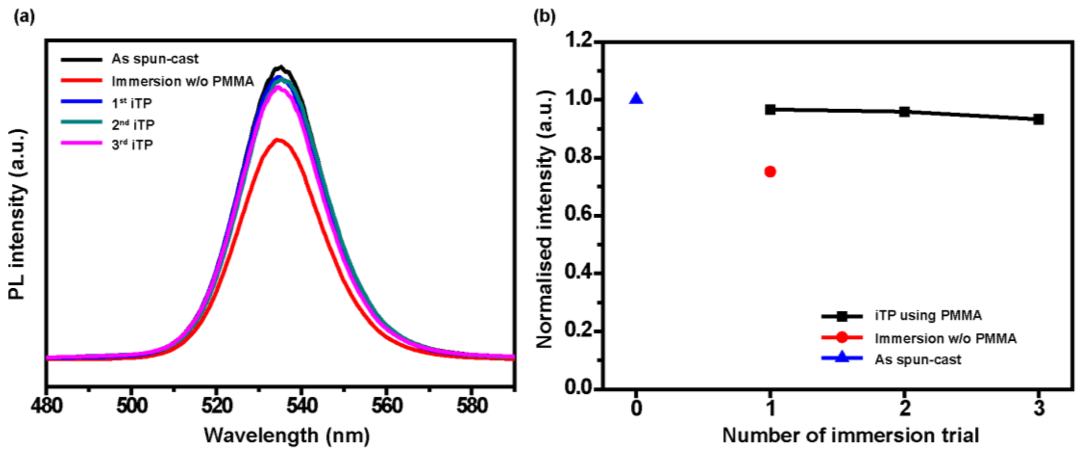

Supplementary Figure 11 | The effect of acetone immersion on the photoluminescence quantum yield of green QDs. (a) Photoluminescence (PL) spectra of green QD films of a reference as spun-cast sample, a spun-cast sample after single immersion, and iTP samples after repeated immersion cycles. **(b)** PL quantum yield of green QD films (as spun-cast film is a reference) plotted after repeated iTP trials.

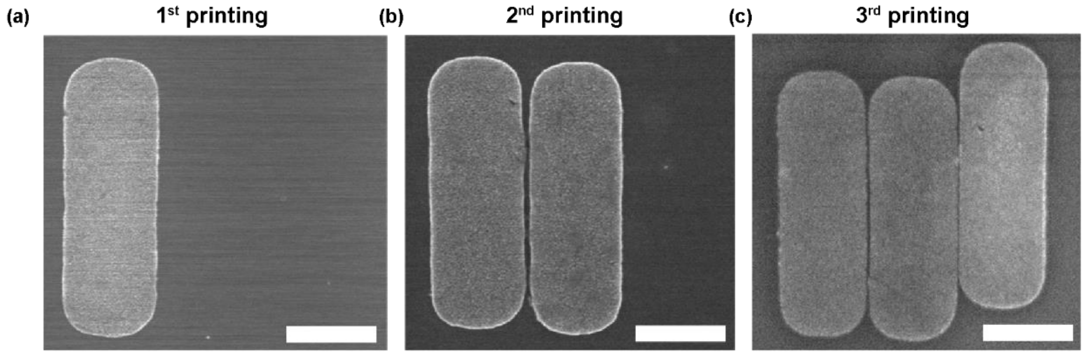

Supplementary Figure 12 | SEM images of sequentially printed QD pixels. (a) 1st printing, **(b)** 2nd printing, **(c)** 3rd printing. The scale bars denote 500 nm.

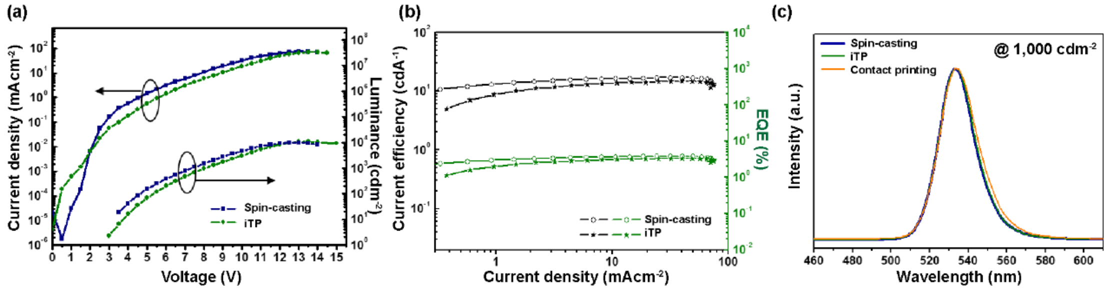

Supplementary Figure 13 | Device performance of ELQLED fabricated by spin-casting (incapable of patterning) and iTP. (a) Current density-voltage-luminance (*J-V-L*) characteristics of ELQLED devices fabricated using spin-casting and iTP. **(b)** Current efficiency and external quantum efficiency (*CE-EQE*) characteristics of ELQLED devices fabricated using spin-casting and iTP. **(c)** Normalized electroluminescence (EL) spectra of ELQLED devices fabricated using spin-casting, iTP, and contact printing.

(a)

	Spin-casting	Contact printing	iTP
**Turn-on-voltage (V)**	2.5	3.3	2.7
**Wavelength peak (nm, @ 1,000 cdm^{-2})**	533.0	534.0	533.5
**Maximum luminance (cdm^{-2})**	9899.3	3525.4	10711.0

(b)

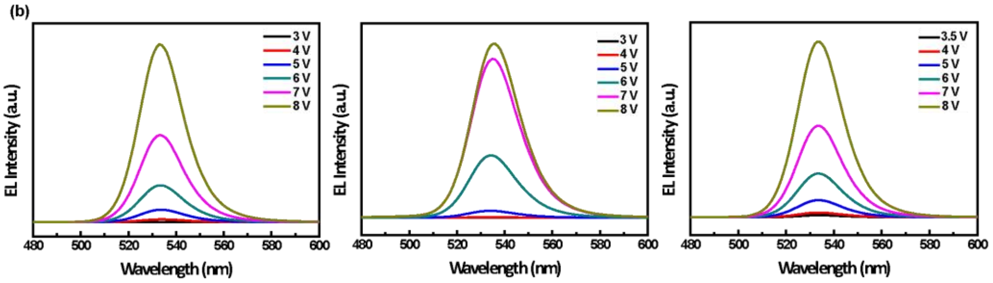

(c)

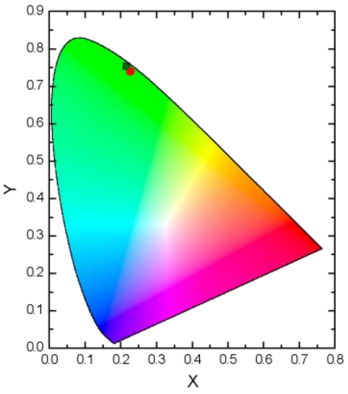

CIE coordinate (@ 1,000 cdm^{-2})	X	Y
**Spin casting**	0.215	0.754
**Contact printing**	0.227	0.739
**iTP**	0.216	0.753

Supplementary Figure 14 | Summary of EL characteristics of spun-cast, contact-printed and iTP devices. (a) Summarized EL characteristics of spun-cast, contact-printed, and iTP devices. **(b)** Voltage-EL spectra of spun-cast, contact-printed, and iTP devices. **(c)** Colour coordinates of spun-cast, contact-printed, and iTP devices.

Supplementary Table 1 | Refractive indices and dielectric constant values used to calculate the *A* values of solid/liquid/air components.

Material	Refractive index *n*	Dielectric constant *ε* ($n_i^2 = \varepsilon_i$ visible spectral range)	REF
Si	3.97660	15.8130	[1]
SiO_x	1.46000	2.13160	[2,3]
PDMS	1.42600	2.03350	[4]
Air	1.00028	1.00056	[5]
Heptane	1.38720	1.92430	[6]
Toluene	1.49680	2.24040	[7]
Chloroform	1.44410	2.08540	[7]

Supplementary Table 2 | Calculated *A* values of each solvent composition with regard to different solid layers.

Solvent	Solid layer	Calculated Hamaker constant $A_{Solvent/Solid}$ (d_{PDMS} = 3 nm, d_{SiOx} = 1.7 nm)
Heptane (H)	PDMS	$A_{H/PDMS} = -4.2491 \times 10^{-21}$
	SiO_x	$A_{H/SiOx} = -7.9179 \times 10^{-21}$
	Si	$A_{H/Si} = -1.5931 \times 10^{-19}$
Toluene (T)	PDMS	$A_{T/PDMS} = 9.4521 \times 10^{-21}$
	SiO_x	$A_{T/SiOx} = 4.8841 \times 10^{-21}$
	Si	$A_{T/Si} = -1.9258 \times 10^{-19}$
Chloroform (C)	PDMS	$A_{C/PDMS} = 2.2143 \times 10^{-21}$
	SiO_x	$A_{C/SiOx} = -1.9328 \times 10^{-21}$
	Si	$A_{C/Si} = -1.7724 \times 10^{-19}$

Supplementary Table 3 | Summary of resolution and device performance information of various printing based ELQLEDs. The resolution and device performance of ELQLEDs fabricated in this work is marked in red.

Year	Printing technology	Feature width and pattern resolution	Device performances<br>Red(R), Green(G), Blue(B), White(W)	REF
2008	Contact-printing<br>QD spin-coating on PDMS (Parylene-C modified)	Two-colour: 25 μm<br>1,000 PPI	EQEs: 1.0% (R), 0.5% (G), 0.2% (B)	8
2008	Microcontact-printing<br>QD spin-coating on PDMS (SU-8 modified)	No patterning,<br>single QD film	Maximum current efficiency (Max CE): 0.4 cdA^{-1}<br>Luminance (Lum): 256 cdm^{-2} at 12 V (W)	9
2011	Transfer-printing<br>QD pick-up from donor substrate by kinetic control	Single colour: 300 nm<br>Full-colour: ~ 100 μm<br>~ 100 PPI	Max Lum: 16,380 cdm^{-2} (R), 6,425 cdm^{-2} (G), 423 cdm^{-2} (B)<br>Max CE: ~ 4 cdA^{-1} (R), ~ 0.5 cdA^{-1} (G), ~ 0.04 cdA^{-1}	10
2013	Transfer-printing<br>QD pick-up from donor substrate aided by lifting layer	Single colour: ~ 100 μm	Max Lum: 3,380 cdm^{-2}<br>Max CE: 0.44 cdA^{-1} (W)	11
2015	Intaglio transfer-printing<br>QD pick-up from donor substrate using intaglio trench	Full-colour: 6 μm<br>2,460 PPI	Lum: 14,000 cdm^{-2} at 7 V<br>EQE: 2.35% at 4.5 V (W)	12
**This work**	**Immersion transfer-printing**<br>**Direct patterning of QDs followed by pressure-free transfer**	**Full-colour: 500 nm**<br>**14,063 PPI**	**Max Lum: 10711 cdm^{-2} (G)**<br>**Max CE: 14.8 cdA^{-1} (G)**<br>**Max EQE: 3.3% (G)**	-

Supplementary Note

Supplementary Note 1 | Theoretical and experimental consistency

A heptane/toluene (8.5:1.5 heptane to toluene ratio) based QD solution was spun-cast on a flat Si/SiO_x/PDMS substrate, and a partially dewetted morphology of the dried QD film was obtained. (Fig. 2(c), left inset) To analyze the feasibility of spinodal dewetting morphology, the consistency between the calculated and experimental characteristic wavelength λ_s (the periodicity of unstable modes whose amplitude grows fastest) values was investigated. The spinodal characteristic wavelength λ_s can be expressed as:

$$\lambda_s(h) = \sqrt{\frac{-8\pi^2\sigma_{lv}}{\Phi''(h)}}$$

The λ_s of the Si/Sio_x/PDMS/toluene/air regime was plotted as a function of film thickness h in Fig. 2(c). The left inset shows an AFM image of the experimentally obtained dewetted film whose FFT image is shown in the right. The analyzed λ_s of 1.3 μm was obtained according to the FFT image, which is consistent with the theoretically acceptable range of λ_s of toluene dewetting, that is, below a few micrometers. We can put the experimentally observed λ_s in the above function (Equation 4) and the corresponding critical film thickness (4.7 nm) will then be obtained. Because the actual solution used in the experiment has a heptane/toluene binary solvent composition, it is feasible that the estimated h_{crit} (4.7 nm) is smaller than the calculated h_{crit} of the toluene-only solvent (5.63 nm).

Supplementary References

1 Aspnes, D. E. & Studna, A. A. Dielectric Functions and Optical-Parameters of Si, Ge, Gap, Gaas, Gasb, Inp, Inas, and Insb from 1.5 to 6.0 Ev. *Phys Rev B* **27**, 985-1009, doi:DOI 10.1103/PhysRevB.27.985 (1983).

2 Chen, Y. Y. & Jin, G. Refractive index and thickness analysis of natural silicon dioxide film growing on silicon with variable-angle spectroscopic ellipsometry. *Spectroscopy-Us* **21**, 26-+ (2006).

3 Ma, H. P. *et al.* Systematic Study of the SiOx Film with Different Stoichiometry by Plasma-Enhanced Atomic Layer Deposition and Its Application in SiOx/SiO2 Super-Lattice. *Nanomaterials-Basel* **9**, doi:ARTN 5510.3390/nano9010055 (2019).

4 Schneider, F., Draheirn, J., Kamberger, R. & Wallrabe, U. Process and material properties of polydimethylsiloxane (PDMS) for Optical MEMS. *Sensor Actuat a-Phys* **151**, 95-99, doi:10.1016/j.sna.2009.01.026 (2009).

5 Ciddor, P. E. Refractive index of air: New equations for the visible and near infrared. *Appl Optics* **35**, 1566-1573, doi:Doi 10.1364/Ao.35.001566 (1996).

6 Kerl, K. & Varchmin, H. Refractive-Index Dispersion (Rid) of Some Liquids in the Uv/Vis between 20-Degrees-C and 60-Degrees-C. *J Mol Struct* **349**, 257-260, doi:Doi 10.1016/0022-2860(95)08758-N (1995).

7 Kedenburg, S., Vieweg, M., Gissibl, T. & Giessen, H. Linear refractive index and absorption measurements of nonlinear optical liquids in the visible and near-infrared spectral region. *Opt Mater Express* **2**, 1588-1611, doi:Doi 10.1364/Ome.2.001588 (2012).

8 Kim, L. *et al.* Contact Printing of Quantum Dot Light-Emitting Devices. *Nano Lett* **8**, 4513-4517, doi:10.1021/nl8025218 (2008).

9 Rizzo, A., Mazzeo, M., Biasiucci, M., Cingolani, R. & Gigli, G. White Electroluminescence from a Microcontact-Printing-Deposited CdSe/ZnS Colloidal Quantum-Dot Monolayer. *Small* **4**, 2143-2147, doi:10.1002/smll.200800350 (2008).

10 Kim, T. H. *et al.* Full-colour quantum dot displays fabricated by transfer printing. *Nat Photonics* **5**, 176-182, doi:10.1038/Nphoton.2011.12 (2011).

11 Kim, T. H. *et al.* Heterogeneous stacking of nanodot monolayers by dry pick-and-place transfer and its applications in quantum dot light-emitting diodes. *Nat Commun* **4**, doi:ARTN 263710.1038/ncomms3637 (2013).

12 Choi, M. K. *et al.* Wearable red-green-blue quantum dot light-emitting diode array using high-resolution intaglio transfer printing. *Nat Commun* **6**, doi:ARTN 714910.1038/ncomms8149 (2015).
