# Supplementary material for: Thermodynamic-driven polychromatic quantum dot patterning for light-emitting diodes beyond eye-limiting resolution
Source: Nat Commun. 2020 Jun 16;11:3040. doi: 10.1038/s41467-020-16865-7 (PMC7297963; doi:10.1038/s41467-020-16865-7)
Supplement: Supplementary file 3 — Description of Additional Supplementary Files [file 41467_2020_16865_MOESM3_ESM.docx]

**Description of Additional Supplementary Files**

File Name: Supplementary Movie 1

Description: The movie clip shows the customised alignment demonstration of the 3 µm QD pixel arrays during the sequential printing step of the immersion transfer printing.
